# Juglone triggers apoptosis of non-small cell lung cancer through the reactive oxygen species -mediated PI3K/Akt pathway

**DOI:** 10.1371/journal.pone.0299921

**Published:** 2024-05-30

**Authors:** Jian Zhong, Yongzhi Hua, Shuting Zou, Bo Wang

**Affiliations:** 1 Affiliated Nanjing Brain Hospital, Nanjing Medical University, Nanjing, Jiangsu, PR China; 2 Digestive Department, Nanjing Lishui District Hospital of Traditional Chinese Medicine, Nanjing, Jiangsu, PR China; University of Michigan, UNITED STATES

## Abstract

Non-small cell lung cancer (NSCLC) is one of the most common malignancies worldwide, and oxidative stress plays a crucial role in its development. Juglone, a naturally occurring naphthoquinone in *J*. *mandshurica*, exhibits significant cytotoxic activity against various cancer cell lines. However, whether the anticancer activity of juglone is associated with oxidative stress remains unexplored. In this study, mouse Lewis lung cancer (LLC) and human non-small cell lung cancer A549 cells were used to explore the anticancer mechanisms of juglone. Juglone inhibited LLC and A549 cells viability, with IC50 values of 10.78 μM and 9.47 μM, respectively, for 24 h, and substantially suppressed the migration and invasion of these two lung cancer cells. Additionally, juglone arrested the cell cycle, induced apoptosis, increased the cleavage of caspase 3 and the protein expression of Bax and Cyt c, and decreased the protein expression of Bcl-2 and caspase-3. Furthermore, juglone treatment considerably increased intracellular reactive oxygen species (ROS) and malondialdehyde (MDA) levels, but suppressed glutathione peroxidase 4 (GPX4) and superoxide dismutase (SOD) activities. It also inhibited the phosphatidylinositol 3-kinase (PI3K)/Akt signaling pathway, which was attenuated by 1,3-diCQA (an activator of PI3K/Akt). Moreover, N-acetylcysteine (a ROS scavenger) partially reversed the positive effects of juglone in terms of migration, invasion, ROS production, apoptosis, and PI3K/Akt pathway-associated protein expression. Finally, in tumor-bearing nude mouse models, juglone inhibited tumor growth without any apparent toxicity and significantly induced apoptosis in NSCLC cells. Collectively, our findings suggest that juglone triggers apoptosis via the ROS-mediated PI3K/Akt pathway. Therefore, juglone may serve as a potential therapeutic agent for the treatment of NSCLC.

## 1. Introduction

Lung cancer accounts for 11.4% of recently reported cancerous patients, and causes more than 1.8 million lung cancer-related deaths globally in 2020 [1]. Despite significant advances in diagnosis and treatment, the prognosis for lung cancer remains poor, with a five-year survival rate of approximately 20% [2]. Non-small cell lung cancer (NSCLC) accounts for more than 80% of all patients with lung cancer [3]. Currently, the best treatment for NSCLC is surgical resection; however, this method is accompanied by a high risk of postoperative recurrence and reduced survival rates. Additionally, low efficiency, adverse side effects, drug resistance, and high costs limit the benefits of basic anticancer strategies (chemotherapy and radiotherapy) and emerging therapies (immunotherapy and molecular targeted therapy). Consequently, it is urgent to develop novel anticancer agents, with the characteristics of low biological toxicity, high efficiency and low price, for the treatment of patients with NSCLC.

Apoptosis is a process of programmed cell death process and plays a vital role in maintaining homeostasis and growth through removing unnecessary cells. Many cellular events, such as mitochondrial damage, death receptor expression, and excessive reactive oxygen species (ROS) production, contribute to apoptotic signals [4]. Moreover, apoptotic cell death is closely associated with caspase activation, which plays a crucial role in the chemotherapy for multiple cancers [5]. Notably, numerous studies have demonstrated that induction of apoptosis can effectively inhibit the spread of cancers, including lung, liver, and colon cancers [6–8]. Furthermore, as ROS mediate the phosphatidylinositol-3-kinase (PI3K)/Akt (protein kinase B) pathway, some researchers have demonstrated that the inhibition of PI3K and Akt could negatively regulate the activity of Cytochrome c (Cyt c) and caspase-3, which are beneficial for cell proliferation inhibition and apoptosis induction [9, 10]. Concordantly, Wang *et al*. confirmed that alnustone effectively inhibited hepatocellular carcinoma BEL-7402 and HepG2 cell growth via the ROS-mediated PI3K/Akt/mTOR/p70S6K pathway [11].

Juglone (5-hydroxy-1,4-naphthoquinone) is a natural phenolic compound isolated from green walnut husks of *J*. *mandshurica*, which is a deciduous tree widely distributed in China, the USA, Iran, and India [12]. The chemical composition of the green walnut husks of *J*. *mandshurica* is complex and mainly comprises naphthoquinones, flavonoids, diarylheptane, triterpenoids, fatty alcohols, phenolic acids, and polysaccharides [13, 14]. Notably, naphthoquinone compounds are considered to be the main anticancer active components of this species. Juglone possesses various pharmacological activities, including anti-microbial, anticancer, anti-inflammatory, immunomodulatory, anti-fungal properties, as well as apoptotic and anti-angiogenesis properties [15–17]. The antioxidant characteristics of phenolic compounds help combat oxidative stress, thereby preventing the development of multiple diseases and aging. It was reported that a small amount of juglone quickly oxidized a large amount of reduced nicotinamide adenine dinucleotide phosphate, leading to remarkable increase of oxygen consumption and ROS production. Therefore, as a quinone molecule, juglone acts as a redox cycling agent and is beneficial for ROS production [18]. Juglone exerts broad anticancer activity against human leukemia, cervical carcinoma, and pancreatic cancer, both *in vitro* and *in vivo* [15, 19, 20]. The apoptotic caspase cascade activation and ROS accumulation are the possible molecular mechanisms of action [21]. 2-Methoxy-6-acetyl-7-methyljuglone (MAM), a natural naphthoquinone, induces NO-dependent apoptosis and necroptosis by H2O2-dependent JNK activation in A549 lung cancer cell [22].

In this study, we aimed to explore the anti-NSCLC effects and the associated mechanisms of action of juglone, focusing on LLC and A549 cells *in vitro* and LLC tumor xenografts *in vivo*. Additionally, cell viability, migration, invasion, apoptosis, oxidative stress, and ROS-mediated PI3K/Akt signaling pathways were evaluated.

## 2. Materials and methods

### 2.1. Reagents and drugs

Juglone (purity≥98%) was obtained from Shanghai Yuanye Biotechnology Co., LTD (Shanghai, China). Cis-platinum was purchased from Qilu Pharmaceutical Co., LTD (Jinan, China). The primary antibodies for PI3K, p-PI3K, Akt, p-Akt, caspase-3, cleaved caspase-3, Bcl-2, Bax, Cyt c, and β-actin were obtained from Santa Cruz Biotechnology Inc. (Dallas, CA, USA). 1,3-Dicaffeoylquinin acid (1,3-diCQA, an activator of PI3K/Akt), N-acetyl-L-cysteine (NAC, a ROS inhibitor), 3-(4,5-dimethylthiazol-2-yl)-2,5-diphenyl tetrazolium bromide (MTT), trypsin, DAPI reagents, and sodium dodecyl sulfate (SDS) were purchased from Sigma-Aldrich (St. Louis, MO, USA). Matrigel was obtained from BD Biosciences (San Jose, CA, USA). High glucose DMEM medium and fetal bovine surum (FBS) were provided by Gibco (Grand Island, NY, USA). ROS detection kit was obtained from KeyGen BioTECH (Nanjing, China). Malondialdehyde (MDA) assay kit, glutathione peroxidase 4 (GPX4) assay kit and superoxide dismutase (SOD) assay kit were purchased from Jiancheng Bioengineering Institute (Nanjing, China).

### 2.2. Cell culture

The NSCLC cell lines (LLC and A549) were purchased from the Cell Bank of the Shanghai Institute of Cell Biology (Shanghai, China). The above cells were cultured in high glucose DMEM medium supplement with 10% fetal bovine serum (FBS), 100 U/ml penicillin and 100 μg/ml streptomycin, maintained at 37°C in a humidified atmosphere of 5% CO_2_. The medium was changed once a day, and digested with 0.25% trypsin every three days. The cells at logarithmic growth stage were used for further experimental research.

### 2.3. Cell viability assay

The LLC and A549 cells were seeded in 96-well plates (5×10^3^ cells/well) overnight. After attachment, cells were treated with various concentrations of juglone (1, 2, 4, 8, 16, and 32 μM) for 24 h, 48 h or 72 h, respectively. Then, 50 μl MTT solution was added into each well for 4 h. The supermatant was removed, 100 μL DMSO was added to solubilize the formazan crystals, and the absorbance at 490 nm was measured on a SpectraMax 190 (Molecular Devices, California, Sunnyvale, USA).

### 2.4. Wound healing assay

LLC and A549 cells were seeded in 6-well plates (1×10^6^ cells/well). After cells were grown to 80%-90% confluence, a 200 μl pipet tip was used to scratch wounds. The detached cells and debris were removed and cells were treated with various concentrations of juglone in serum-free medium for 48 h. Scratches were photographed at 0, 24 h and 48 h. The scratch healing rate was determined by following formula: scratch healing rate = [(start scratch width–end scratch width)/ start scratch width] × 100% [23].

### 2.5. Cell invasion assay

LLC and A549 cells in suspension were plated to the upper chamber of the 24-well hanging chambers with or without various concentrations of juglone in serum-free medium (2.5×10^5^ cells/chamber). Subsequently, 0.5 ml DMEM medium containing 10% FBS was added to the lower chamber. After 24 h, the unmigrated cells from the upper chamber were gently scraped with cotton swabs. The invaded cells were fixed with formalin, stained with 0.1% crystal violet. Finally, 5 random fields were photographed (Olympus IX73, Japan) and the software of Image J was performed to count the invaded cells

### 2.6. Apoptosis assay

Apoptosis analysis of LLC and A549 cells after the exposure of juglone was carried out using Annexin V-PI apoptosis kit (Beckman Coulter, USA). In brief, LLC and A549 cells were treated with 2, 4, and 8 μM juglone for 24h, respectively. The cells were collected, washed with cold PBS, and stained with AnnexinV-FITC and PI. The distribution of apoptosis/necrosis cells was quantified by the FACSort Flow Cytometer (BD, San Jose, CA, USA).

### 2.7. Flow cytometric analysis of cell cycle distribution

To analyze the cell cycle distribution affected by juglone, LLC and A549 cells were seeded in 24-well paltes (5×10^4^ cells/well) overnight. Then, cells were treated with different concentrations of juglone for 24 h. Cells were washed with PBS and fixed with 70% chilled ethanol. Single-cell suspensions were incubated with Ribonuclease (RNase) and PI. The FACSort Flow Cytometer (BD, San Jose, CA, USA) was used to analyze the cell cycle distribution.

### 2.8. ROS generation assay

The DHE fluorescent probe was employed to determine the generation of intracellular ROS in NSCLC cells. In brief, LLC and A549 cells were exposed to juglone for 24 h and then incubated with DCFH-DA fluorescent probe for 20 min in the dark. Subsequently, ROS levels were observed and photograohed with a laser scanning confocal microscope (Olympus, FV3000, Tokyo, Japan)

### 2.9. Detection of MDA, GPX4 and SOD

The levels of MDA, GPX4 and SOD were detected using the corresponding commercial kits. All steps were carried out in strict accordance with the instructions provided by the manufacturer.

### 2.10. Western blotting analysis

The harvested LLC and A549 cells were lysed for 30 min in RIPA buffer. After quantification using bicinchoninic-acid assay and denaturation by heating for 5 min at 95°C, an equal amount of proteins (20 μg) was loaded on 10% sodium dodecyl sulfate-polyacrylamide gels and then transferred to polyvinylidene difluoride (PVDF) membranes, which was blocked in 5% bovine serum albumin (BSA). Subsequently, the primary antibodies were added to the membrane at 4°C overnight to detect the specific proteins. Then, PVDF membrane was incubated with horseradish-peroxidase-conjugated secondary antibodies for 1 h, and enhanced chemiluminescence (ECL) reagent was used to visualize the antigen-antibody reaction.

### 2.11. Xenograft experiment

Fifteen male nude mice (BALB/c nude, 5-week-old) were fed with free water and food intake in a standardized environment (room temperature: 23 ± 2°C; relative humidity: 55 ± 5%; light-dark schedule: lights on 8 a.m. to 8 p.m.). This study was carried out in strict accordance with the recommendations in the Guide for the Care and Use of Laboratory Animals of the National Institutes of Health. The protocol was approved by the Animal Ethics Committee of Jiangsu Provincial Academy of Chinese Medicine (AEWC-20230421-296). All mice were sacrificed by cervical dislocation, and all efforts were made to minimize suffering. During the experiment, technicians monitor the animal health and behavior daily to ameliorate suffering in time. 5 × 10^6^ LLC cells in PBS (200 μl) were subcutaneously injected into the right axillary region of every nude mice. Tumor volume was calculated using the formula as follows: volume = (length ×width^2^)/2 [24]. When tumor volume reached approximately 100 mm^3^, a total number of 18 mice were randomly divided into 3 groups as follows: control group, juglone group and cis-platinum group. Mice in the control group were intraperitoneally injected with 0.2 ml normal saline once a day for 14 days. Mice in the juglone group were intraperitoneally injected with 10 mg/kg juglone once a day for 14 days. Mice in the cis-platinum group were intraperitoneally injected with 5 mg/kg cis-platinum twice a week, a total of 4 times. The body weight and tumor volume were recorded every 2 days. After 14 days, all mice were sacrificed by cervical dislocation. Tumor tissues were isolated, weighed and fixed using 10% formalin or stored at -80°C. Additionally, major organs including heart, liver, spleen, lung and kidney were collected and fixed in 10% formalin for toxicity assay using the Hematoxylin and eosin staining.

### 2.12. Hematoxylin and eosin (H&E) staining

After sacrifice, the heart, liver, spleen, lung and kidney tissues of the BALB/c nude mice were excised for histological analysis. Tissues were fixed in 10% formalin and paraffin-embedded. After slicing, hematoxylin and eosin (H&E) staining was performed for observation. The tissue structure was observed in the magnification of 200X under a light microscope.

### 2.13. TUNEL staining for detection of tumor tissue apoptosis

Tumor cell apoptosis in the xenograft tumor tissues was detected using TUNEL technology. Briefly, tumor tissue was embedded in paraffin, sliced and soaked in gradient ethanol for 3 minutes each time. Then, the section was rinsed by PBS and treated with 20 μg/ml proteinase K working solution at 30°C for 20 min. Subsequently, 50 μL TUNEL reaction mixtures (50 μL TDT and 450 μL fluorescein labeled dUTP solution) were added to the samples from treated groups and only 50 μL fluorescein labeled dUTP solution was added to the samples from negative control group, reacted in dark wet box for 1 hour at 37°C. A drop of glycerol was added and the apoptotic cells were photographed under a light microscope.

### 2.14. Statistical analysis

SPSS 26.0 software was employed for statistical analysis. Multiple comparisons were performed with one-way analysis of variance (ANOVA) tests. The results were from at least triple-independent experiments, and presented as mean ± standard deviation (SD). P < 0.05 was considered statistically significant.

## 3. Results

### 3.1. Juglone inhibits the viability of NSCLC cells

**Fig 1:**
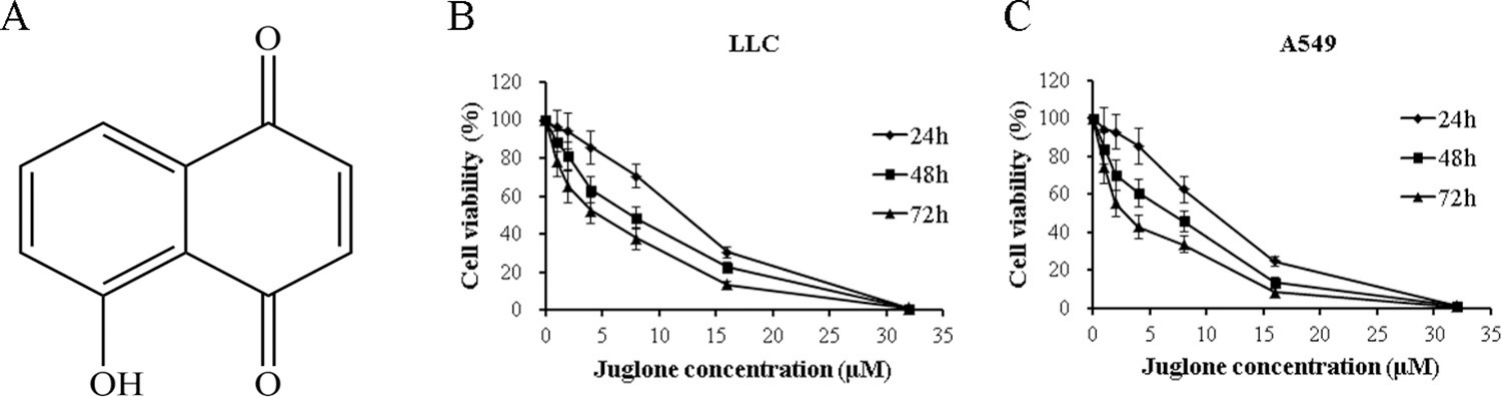
Effect of juglone on the proliferation of different lung cnacer cells by MTT assay. (A) Chemical structure of juglone. (B) Cell viability of LLC cells after treatment with the indicated concentrations of juglone (1, 2, 4, 8, 16, and 32μM) for 24 h, 48 h or 72 h, respectively. (C) Cell viability of A549 cells after treatment with the indicated concentrations of juglone (1, 2, 4, 8, 16, and 32 μM) for 24 h, 48 h or 72 h, respectively. Data were expressed as mean ± SD (n = 3).

LLC and A549 cells were exposed to different concentrations of juglone to explore its survival rates using 3-(4,5-dimethylthiazol-2-yl)-2,5-diphenyltetrazolium bromide (MTT) assay. As shown in Fig 1, juglone inhibited the viability of LLC and A549 cells at increasing concentrations and exposure times. The semi-inhibitory concentration (IC_50_) values of juglone were approximately 10.78 ± 0.98 μM at 24 h, 6.21 ± 0.55 μM at 48 h, and 3.88 ± 0.41 μM at 72 h for LLC cells, and these values were 9.47 ± 1.02 μM at 24 h, 5.01 ± 0.44 μM at 48 h, and 2.82 ± 0.21 μM at 72 h for A549 cells, respectively. Our results suggested that juglone effectively inhibited the growth of NSCLC cells in a concentration- and time-dependent manner.

### 3.2. Juglone inhibits migration and invasion

**Fig 2:**
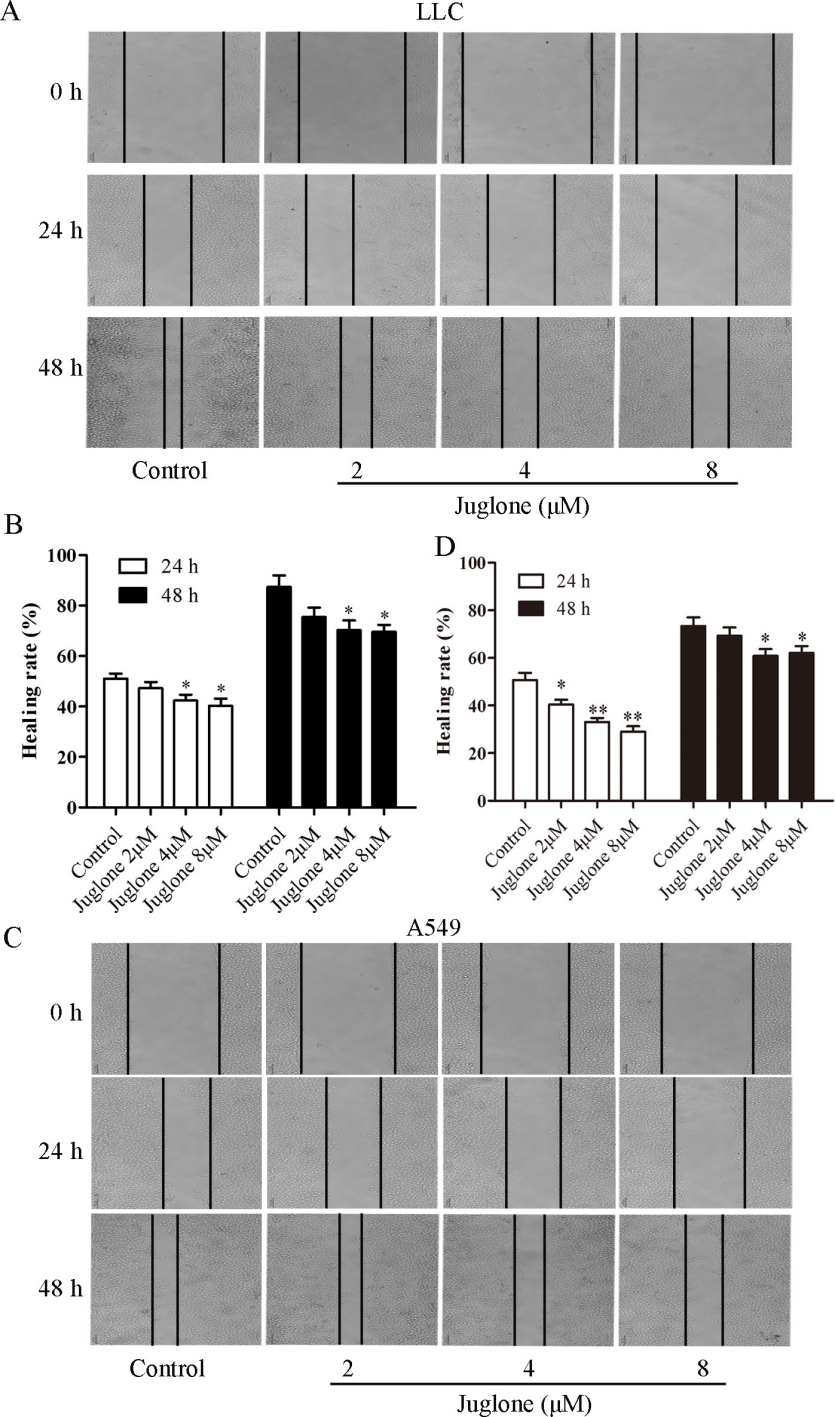
Juglone inhibited the migration of LLC and A549 cells. (A, C) LLC and A549 cells were seeded in six-well plates, then cells were scraped and treated with various concentrations of juglone for 24 h and 48 h. (B, D) Quantification of LLC and A549 cells migration. Data were expressed as mean ± SD (n = 3). *P < 0.05, **p < 0.01, compared with the control group.

**Fig 3:**
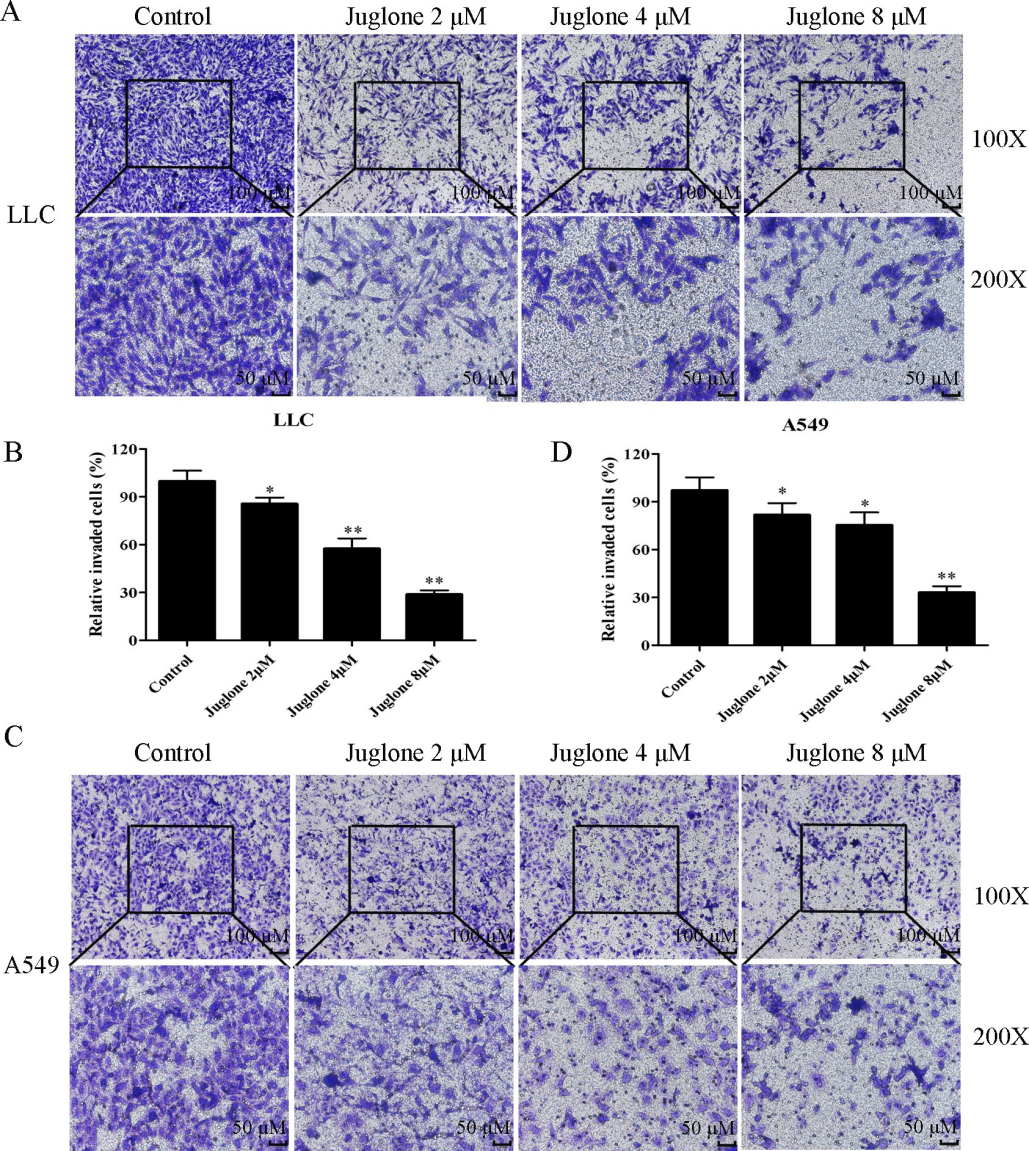
Juglone inhibited the invasion of LLC and A549 cells. (A, C) The invasion of LLC and A549 cells were determined by transwell assay. (B, D) The invaded cells were counted by the software of Image J. Data were expressed as mean ± SD (n = 3). *P < 0.05, **P < 0.01, compared with the control group.

Because of the crucial role of migration and invasion in cancer metastasis, we evaluated the migration and invasion of LLC and A549 cells in juglone-treated groups. The scratch healing rates of LLC cells treated with juglone at 2, 4, and 8 μM for 24 h were (47.32 ± 3.43)%, (42.40 ± 3.27)% and (40.25 ± 4.10)%, respectively, and (51.08 ± 2.80)% in the control group (Fig 2A and 2B). Meanwhile, the scratch healing rates of A549 cells treated with juglone at 2, 4, and 8 μM for 24 h were (40.47 ± 2.83)%, (33.14 ± 2.28)%, and (29.06 ± 3.19)%, respectively, and (50.71 ± 4.29)% in the control group (Fig 2C and 2D). The healing rate was further enhanced when LLC and A549 cells were treated with juglone for 48 h than that for 24 h. The relative number of invaded LLC cells in the 2, 4, and 8 μM juglone-treated groups were all lower than that in the control group (Fig 3A and 3B). Furthermore, the relative number of invaded A549 cells was also significantly suppressed by juglone treatment for 24 h (Fig 3C and 3D). These results indicated that juglone restrained the migration and invasion of LLC and A549 cells.

### 3.3. Effects of juglone on cell cycle and apoptosis

**Fig 4:**
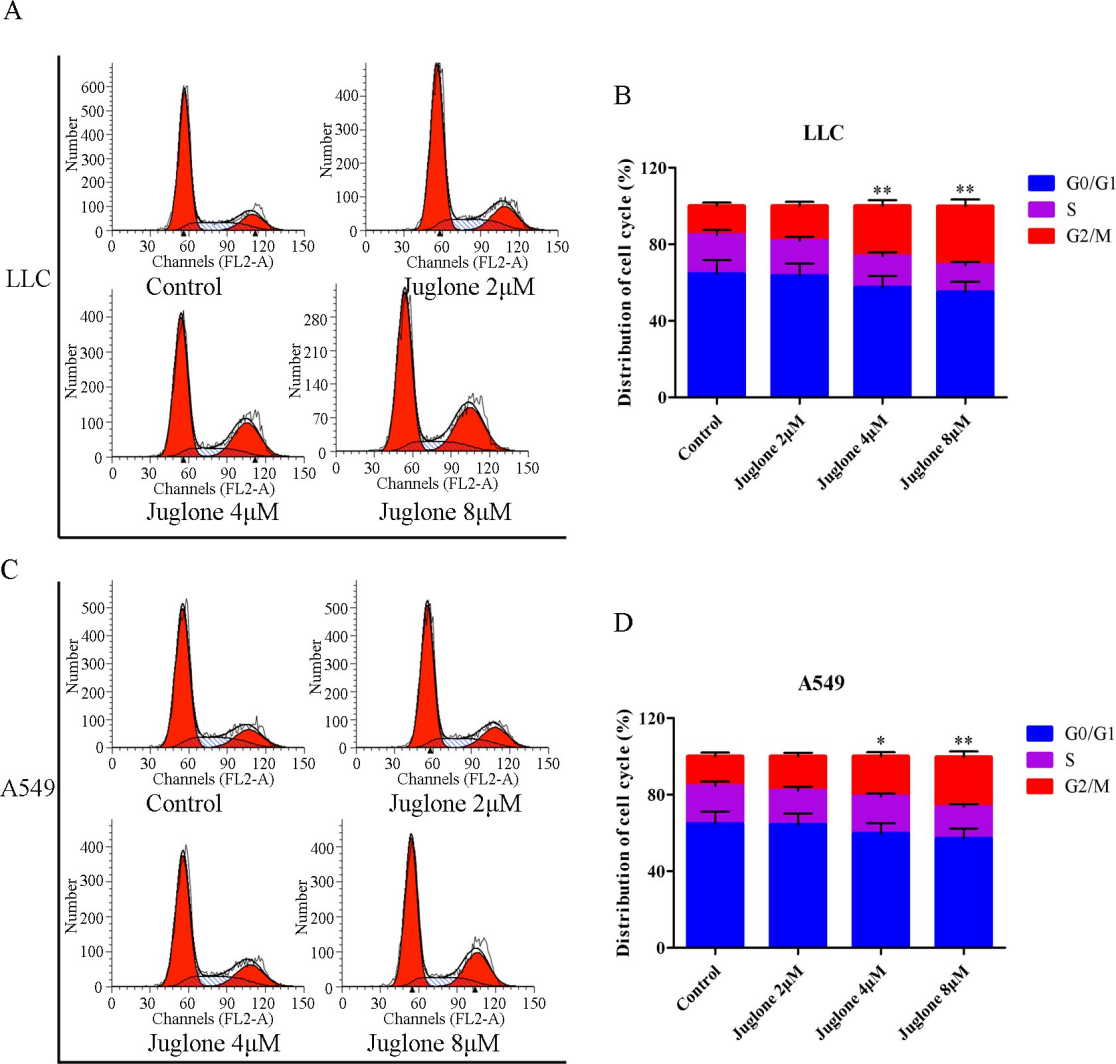
Effect of juglone on cell cycle *in vitro*. (A, B) LLC cell cycle distribution after juglone treatment. (C, D) A549 cell cycle distribution after juglone treatment. Data were expressed as mean ± SD (n = 3). *P < 0.05, **P < 0.01, compared with the control group.

The effect of juglone on cell cycle distribution was assessed by flow cytometry. Juglone disrupts the normal cell cycle of NSCLC cells. As shown in Fig 4, the populations of LLC cells in the G2/M phase were (18.14 ± 2.12)%, (26.02 ± 3.05)%, and (30.87 ± 3.37)% in the 2, 4, and 8 μM juglone treatment groups, respectively, whereas the control group exhibited (15.07 ± 1.76)% cell cycle progession. Similarly, the percentage of A549 cells in the G2/M phase was increased upon treatment with 2, 4, and 8 μM julone, with values of (18.00 ± 1.78)%, (21.01 ± 2.08)% and (26.04 ± 2.96)%, respectively, compared with that in the control group (15.43 ± 1.96)%. These results illustrated that both LLC and A549 cells were arrested in G2/M phase by juglone.

**Fig 5:**
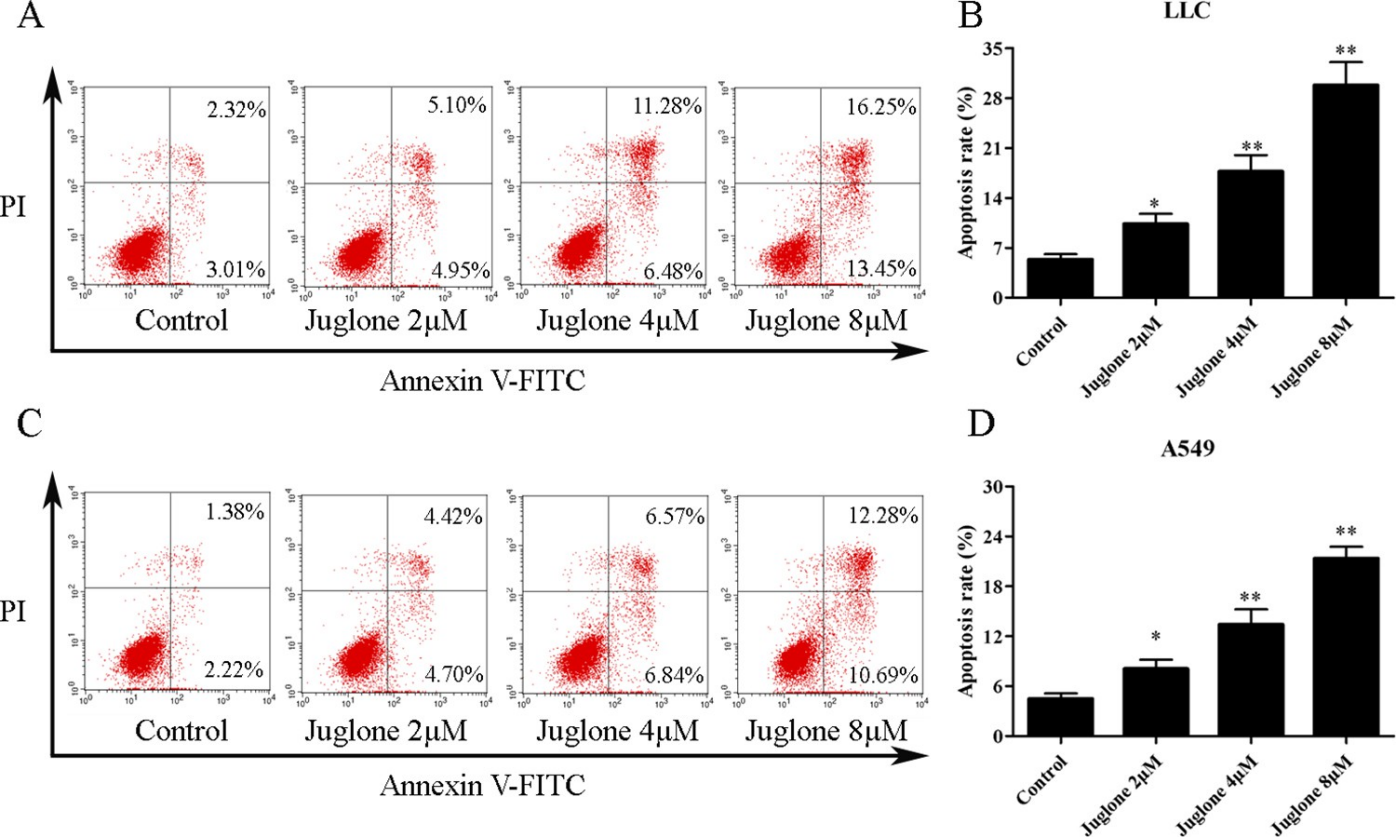
Flow cytometry was performed to evaluate the apoptosis rates of LLC and A549 cells mediated by juglone. (A, B) The apoptosis of LLC cell after juglone treatment. (C, D) The apoptosis of A549 cell after juglone treatment. Data were expressed as mean ± SD (n = 3). *P < 0.05, **P < 0.01, compared with the control group.

**Fig 6:**
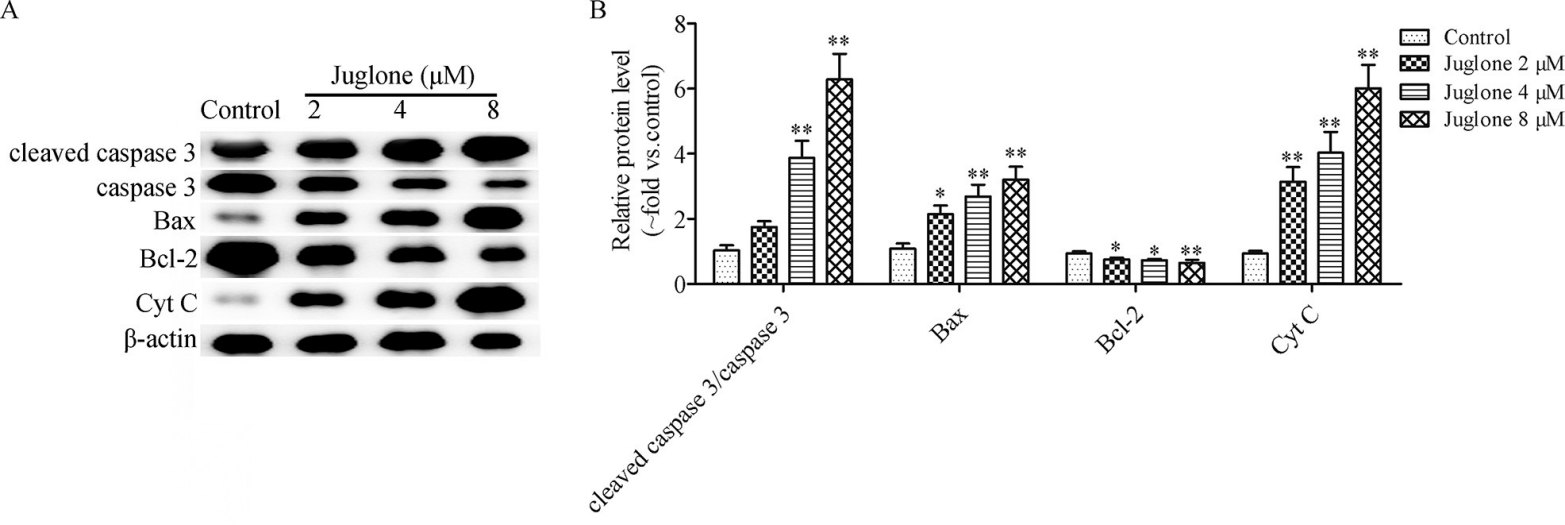
Effect of juglone on the expression of apoptosis-related protein *in vitro*. (A, B) Western blotting analysis of apoptosis-related proteins in LLC cells. Data were expressed as mean ± SD (n = 3). *P < 0.05, **P < 0.01, compared with the control group.

The Annexin V-PI staining was performed to evaluate apoptotic cell death mediated by juglone at the concentrations of 2, 4, and 8 μM. The flow cytometric results indicated that the apoptotic cell population was upregulated from (5.34 ± 0.76)% in the control group to (10.12 ± 1.39)%, (17.01 ± 1.79)%, and (29.90 ± 3.22)% after treatment with juglone at 2, 4, and 8 μM for 24 h, respectively (Fig 5A and 5B). In A549 cells, the apoptosis rate was (4.49 ± 0.64)% in the control group, and this increased to (8.09 ± 1.09)%, (13.42 ± 1.81)%, and (21.36 ± 1.41)% after treatment with juglone at 2, 4, and 8 μM for 24 h, respectively (Fig 5C and 5D). These results suggested that juglone promoted apoptotic cell death in LLC and A549 cells. The western blotting results indicated that juglone increased the ratio of cleaved caspase-3/caspase-3 (P < 0.01), increased the protein expressions of Bax, and Cyt c (P < 0.05, P < 0.01), and decreased the protein expression of Bcl-2 (P < 0.05, P < 0.01) (Fig 6). These findings collectively suggested that juglone induced apoptosis in LLC and A549 cells.

### 3.4. Juglone alters the oxidative stress homeostasis

**Fig 7:**
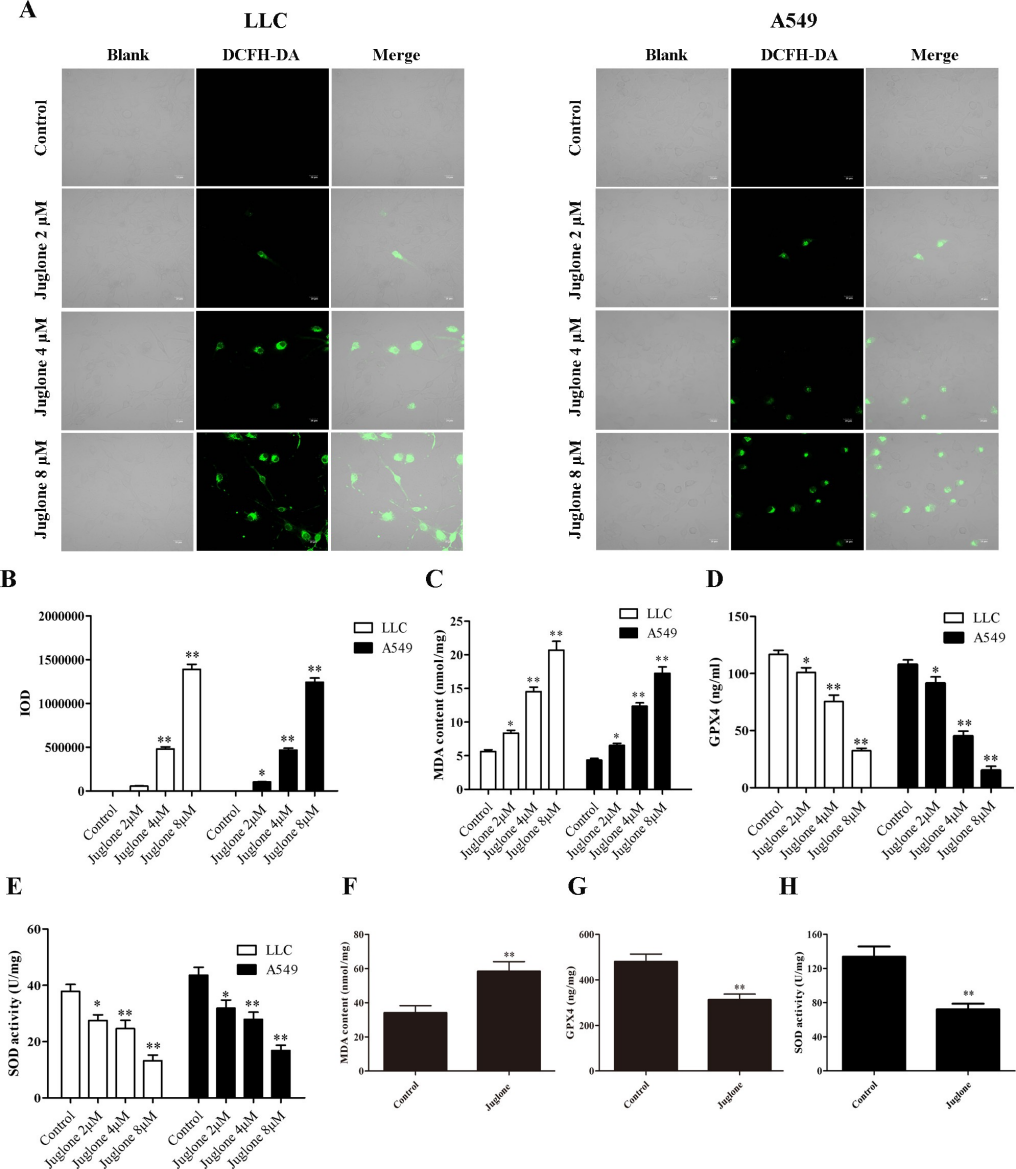
Effect of juglone on oxidative stress *in vitro* and *in vivo*. (A, B) Intracellular ROS production was measured by fluorescence probe method, and analyzed using SPSS software. (C) MDA content *in vitro*. (D) GPX4 level *in vitro*. (E) SOD activity *in vitro*. LLC and A549 cells were treated with juglone (2, 4 and 8 μM) for 24 h. (F) MDA content *in vivo*. (G) GPX4 level *in vivo*. (H) SOD activity *in vivo*. Data were expressed as mean ± SD (n = 3). *P < 0.05, **P < 0.01, compared with the control group.

Apoptosis is typically accompanied by an increase in oxidative stress [25]. The fluorescent probe dichlorodihydrofluorescein diacetate (DCFH-DA) was used to examine the effect of juglone on the production of ROS in LLC and A549 cells. As shown in Fig 7A and 7B, the intracellular ROS levels (green fluorescence intensity) was increased along with the increasing concentration of juglone. Compared with the control group, the fluorescence intensity was significantly increased in juglone at 4 and 8 μM groups in LLC cells (P < 0.01), and the fluorescence intensity was significantly increased in juglone at 2, 4 and 8 μM groups in A549 cells (P < 0.05, P < 0.01). Furthermore, the levels of MDA, GPX4 and SOD, which are associated with oxidative stress, were also determined. Juglone treatment increased MDA level but suppressed GPX4 and SOD activities in lung carcinoma cells compared with that in the control group (Fig 7C–7E). To investigate the effect of juglone on oxidative stress-related indices *in vivo*, MDA level and GPX4 and SOD activities were determined in colonic tissues. The MDA level in the juglone groups was lower than that in the control group, whereas the activity levels of GPX4 and SOD in the juglone group were higher than those in the control group (P < 0.01) (Fig 7F–7H).

### 3.5. Effect of juglone on the expression of PI3K/Akt pathway-associated proteins

**Fig 8:**
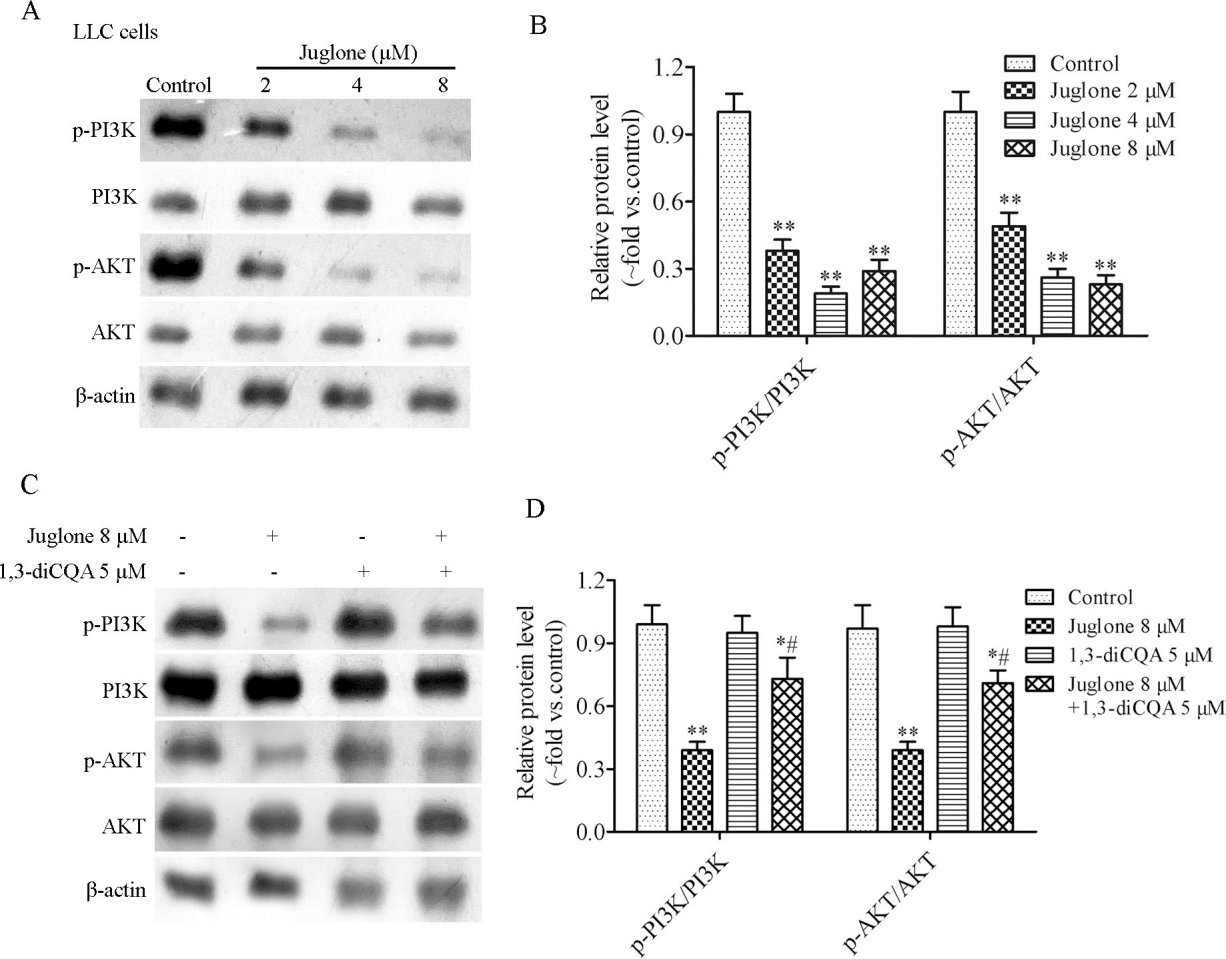
Juglone inhibited the activation of PI3K/Akt pathway *in vitro*. (A, B) The protein expression of PI3K, p-PI3K, Akt and p-Akt were determined by western blotting assay after LLC cells treatment with different concentrations of juglone for 24 h. (C, D) The protein expression of PI3K, p-PI3K, Akt and p-Akt were determined by western blotting assay after LLC cells treatment with juglone (8 μM) and 1,3-diCQA (5 μM) alone or in combination for 24 h. Data were expressed as mean ± SD (n = 3). *P < 0.05, **P < 0.01, compared with the control group; ^#^P < 0.05, compared with the juglone 8 μM group.

The PI3K/Akt pathway is closely associated with carcinogenesis and apoptosis [26]. As shown in Fig 8A and 8B, juglone reduced the ratios of p-PI3K/PI3K and p-Akt/Akt in LLC cells. Furthermore, the activator (1,3-diCQA) of PI3K/Akt was used to determine whether juglone suppressed the PI3K/Akt signaling pathway. LLC cells were exposed to 1,3-diCQA and juglone, alone or in combination, for 24 h. The results indicated that 1,3-diCQA attenuated the effect of juglone on PI3K/Akt pathway-related protein expression (Fig 8C and 8D).

### 3.6. ROS scavenger partially reverses the inhibitory effects of juglone on the PI3K/Akt pathway

**Fig 9:**
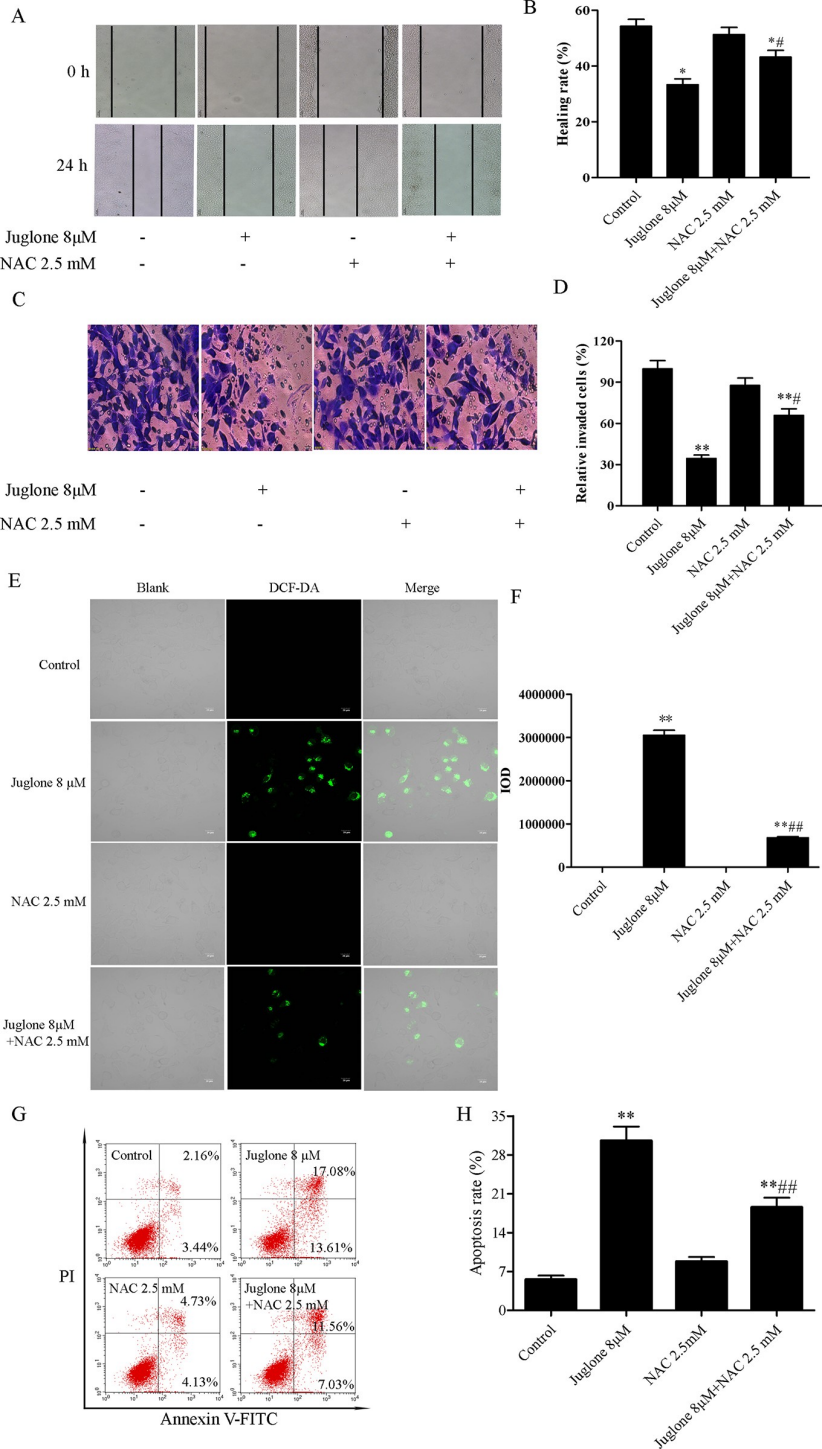
NAC attenuated juglone-mediated apoptosis. (A, B) Wound healing assay and quantification of migration. (C, D) Cell invasion assay and quantification of invaded cells. (E, F) DCF-DA staining was performed to determine ROS production in LLC cells, and analyzed using SPSS software. (G, H) Flow cytometry was carried out to evaluate the cellular apoptosis, and the quantification for apoptosis in LLC cells was analyzed. Data were expressed as mean ± SD (n = 3). *P < 0.05, **P < 0.01, compared with the control group; ^#^P < 0.05, ^##^P < 0.01, compared with the juglone 8 μM group.

**Fig 10:**
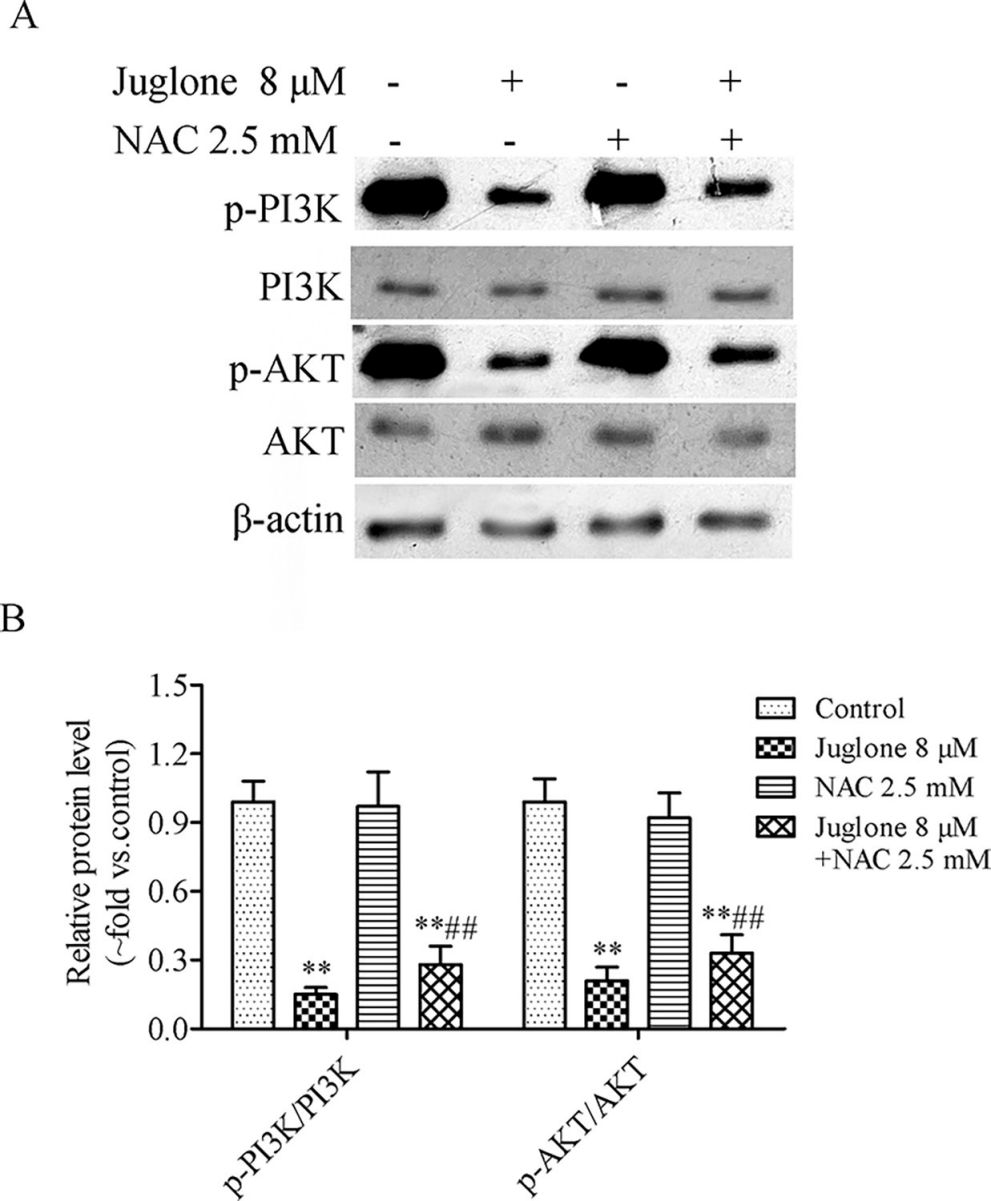
NAC reversed juglone-mediated PI3K/Akt pathway. (A, B) Western blotting analysis and quantification of the PI3K/Akt-related proteins expression in LLC cells. Data were expressed as mean ± SD (n = 3). **P < 0.01, compared with the control group; ^##^P < 0.01, compared with the juglone 8 μM group.

NAC, a well-known ROS scavenger, was used to determine the effects of ROS on juglone-stimulated apoptosis. LLC cells were cotreated with NAC or juglone to explore the relationship between juglone-mediated ROS production and apoptosis. Wound healing and cell invasion assays suggested that NAC could evidently reverse the inhibitory effects of juglone on the migration and invasion of LLC cells. Notably, when the cells were treated with a combination of juglone (8 μM) and NAC (2.5 mM), the scratch healing rates increased from (33.34 ± 2.96)% to (43.22 ± 3.39)%, and the relative number of invaded cells increased from (34.45 ± 3.48)% to (65.80 ± 6.92)% compared with that during treatment with juglone alone (8 μM) (Fig 9A–9D). The DCFH-DA staining indicated that juglone-induced ROS production was significantly abolished when LLC cells were co-treated with juglone and NAC (P< 0.01) (Fig 9E and 9F). The apoptosis rate in the juglone only group and juglone and NAC-cotreated group was (30.54 ± 2.55)% and (18.56 ± 1.74)%, respectively. Additionally, the percentage of apoptotic cells in the NAC group was similar to that in the control group (Fig 9G and 9H). Subsequently, western blot analysis was performed to determine whether ROS participate in the regulation of the PI3K/Akt pathway. As a result, the ratios of p-PI3K/PI3K and p-Akt/Akt were observed to be 0.15 ± 0.03 and 0.21 ± 0.06 in juglone-treated LLC cells. However, the ratios of p-PI3K/PI3K and p-Akt/Akt were markedly upregulated when LLC cells were treated with a combination of juglone and NAC (Fig 10A and 10B). These results indicate that NAC partially attenuated the suppression of juglone on the protein expression associated with the PI3K/Akt pathway. Taken together, these results indicate that ROS production is essential for the apoptosis and suppression of the PI3K/Akt pathway induced by juglone on NSCLC cells.

### 3.7. Juglone inhibits NSCLC cell growth in vivo

**Fig 11:**
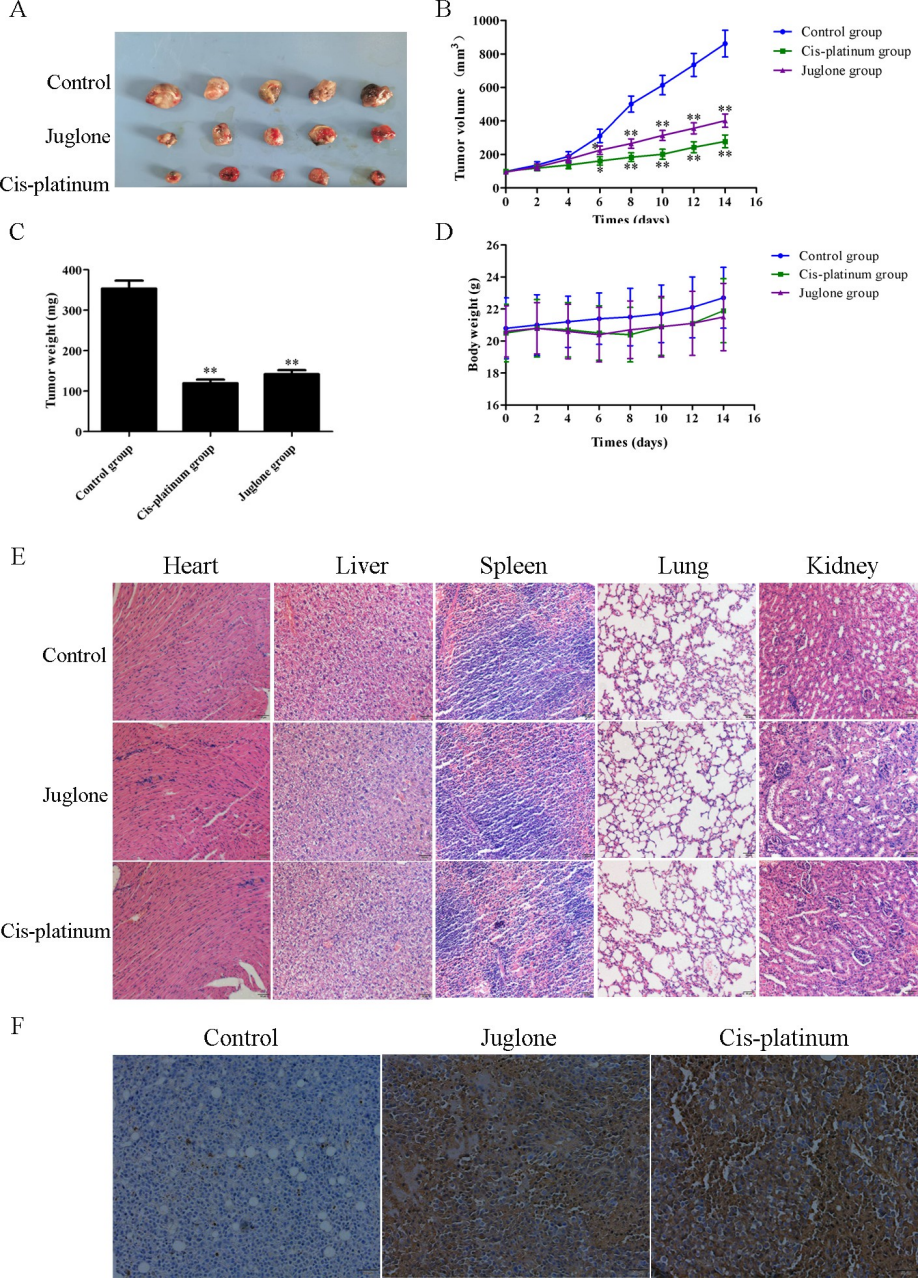
Juglone inhibited tumor growth and induced tumor tissue apoptosis in LLC-bearing mice. (A) Juglone restrained tumor growth in the subcutaneous xenograft model. (B) Tumor volume. (C) Tumor weight. (D) Body weight. (E) H&E staining of tissues from heart, liver, spleen, lung, and kidney (200X). (F) Apoptosis analysis of LLC tumor tissues by TUNEL assay (200X). Data were expressed as mean ± SD (n = 3). *P < 0.05, **P < 0.01, compared with the control group.

To demonstrate the anticancer effect of juglone, *in vivo* experiments were performed using an athymic nude mouse model with LLC cells, followed by an intraperitoneal injection of juglone. As presented in Fig 11A–11C, the tumor volume and weight were remarkably decreased in mice receiving juglone. In contrast, the body weight was not significantly altered among the control, juglone, and cis-platinum groups over the experimental period (Fig 11D), partly suggesting that juglone (10 mg/kg) has limited side effects *in vivo*. To estimate the potential toxicity of juglone, H&E staining of the main visceral organs of BALB/c nude mice was performed. A comparison of the H&E pictures of the heart, liver, spleen, lung, and kidney from the control, juglone, and cis-platinum groups indicated that no distinct pathological lesions during these three groups (Fig 11E). Juglone markedly induced apoptosis in the tumor tissues of LLC cells, as determined by the TUNEL assay (Fig 11F). The above results indicate that juglone effectively inhibits tumor growth and induces apoptosis in NSCLC cells *in vivo*.

## 4. Discussion

Owing to the lack of effective therapeutic methods, there is an urgent need to identify novel potential anticancer drugs for NSCLC treatment. Juglone, a naphthoquinone compound, exerts cytotoxic effects on multiple tumor cells. For example, juglone induces apoptosis by inhibiting the expression of inflammatory molecules in colon cancer cells [27]. We also observed that juglone exerted potent cytotoxicity in NSCLC cells. In this study, juglone treatment restrains the migration and invasion in both LLC and A549 cells. These results indicate that juglone has an inhibitory effect on NSCLC cells metastasis.

Apoptosis is the primary and most well-explored form of programmed cell death, and is triggered by extrinsic and intrinsic apoptotic pathways [4]. Mitochondrial dysfunction, including an impaired mitochondrial electron transport chain, mitochondrial membrane potential depolarization, and ROS generation, is the primary mechanism underlying mitochondria-mediated apoptosis [28]. Oxidative stress stimulates Bax and Bcl-2 molecules to insert into the outer membrane of the mitochondria, causing Cyt c to be released into the cytoplasm, which then binds to caspase-9 and other molecules [29]. Caspase-9 activation activates the caspase cascade by processing caspases-3 and -7, ultimately inducing cell apoptosis [30]. The Bcl-2 family of proteins plays a critical role in the intrinsic apoptotic pathway, and the ratio of pro-apoptotic Bax to anti-apoptotic Bcl-2 protein is an indispensable indicator to determine whether mitochondrial apoptosis occures [31]. Moreover, caspase-3, an enzyme associated with apoptosis induction, is widely recognized in this process [32]. Consequently, targeting apoptosis is a promising strategy for the discovery and development of novel anticarcinogenic agents. In line with our study findings, the activation of caspase-3 by juglone treatment was observed. Furthermore, the determination of mitochondrial protein Cyt c level following exposure to juglone strengthens the evidence suggesting that juglone triggers apoptosis through the intrinsic apoptotic pathway in NSCLC cells. Our findings are consistent with those of previous studies, which indicated that juglone induced apoptosis via the intrinsic apoptotic pathways in the human glioblastoma multiforme and melanoma [17, 33].

The PI3K/Akt pathway is a crucial signaling pathway responsible for promoting protein synthesis and is considered a regulatory factor in cell proliferation, differentiation, and migration [34]. The PI3K/Akt pathway is usually over-activated and plays pro-survival and anti-apoptotic roles in various malignant tumors [35]. Therefore, targeting on the oncogenic PI3K/Akt signaling pathway is regarded as an extremely valuable strategy for anticancer drug development. Concordantly, Zuo *et al*. demonstrated that voacamine inhibited the PI3K/Akt signaling pathway to suppress breast cancer progression [36]. Similarly, Liu *et al*. suggested that zoledronic acid enhanced the anti-tumor effect of cisplatin in orthotopic osteosarcoma via ROS-PI3K/Akt signaling [37]. Consistent with these findings, our results demonstrated that juglone treatment inhibited the phosphorylation of PI3K and Akt in both LLC and A549 cells. Additionally, the effect of juglone on the expression of proteins associated with the PI3K/Akt pathway was markedly attenuated by 1,3-diCQA (a PI3K/Akt activator). These results collectively highlight that the PI3K/Akt pathway is crucial in mediating juglone-induced apoptosis in NSCLC cells.

ROS accumulation-induced oxidative stress serves as a pivotal criterion for initiating cell death in cancer chemotherapy, primarily through the induction of apoptosis [38]. Intracellular antioxidant enzymes (SOD and GPX4) and metabolites (GSH) predominantly participated in the cellular redox defense mechanisms against oxidative stress by scavenging ROS [39]. Therefore, inhibition of these antioxidant molecules encourages intracellular ROS accumulation, consequently causing oxidative stress. Because of the intramolecular hydrogen bonds between its hydroxyl and keto groups and its ability to donate a hydrogen_atom, juglone may serve as an oxidation accelerator or oxidation inhibitor [40]. Some studies have suggested that juglone promoted ROS production, whereas others have reported its antioxidant characteristics [41]. In this study, we observed that juglone treatment increased ROS and MDA levels (an indicator of lipid peroxidation) and reduced SOD activity and GPX4 level. To confirm the causal relationship between juglone-induced ROS production and apoptotic activity, LLC cells were cotreated with juglone and a ROS scavenger (NAC). Consequently, we observed that the inhibitory effects of juglone on cell viability, migration and invasion were attenuated by NAC treatment. Furthermore, we observed that apoptosis was lower in the juglone and NAC-cotreated LLC cells than that in the LLC cells treated with juglone alone. Western blot analysis indicated that the effect of juglone on the expression of apoptosis-associated proteins was attenuated by NAC treatment. Additionally, previous literatures reported that ROS takes part in modulating PI3K/Akt pathway, which was closely related to migration, invasion and apoptosis. Wu *et al*. proved that tormentic acid induced the anti-cancer effect in cisplatin-resistant cervical cancer via ROS-mediated PI3K/Akt pathway [42]. Liu *et al*. demonstrated that sparstolonin B exerted beneficial effects on prostate cancer by acting on the ROS-mediated PI3K/AKT pathway [43]. Juglone could induce ROS generation and suppress the PI3K/Akt pathway, and the inhibitory effect could be partially reversed by NAC (ROS inhibitor). Collectively, these results indicate that the generation of ROS by juglone plays an important role in the apoptosis and acts as an upstream regulator of PI3K/Akt pathway in NSCLC cells, which is consistent with many FDA-approved anti-neoplastic drugs in clinical applications [44].

In conclusion, our study confirmed that juglone inhibited the viability, migration, and invasion of LLC and A549 cells. This mechanism is associated with ROS generation, induction of apoptosis, and inhibition of the PI3K/Akt signaling pathway. Additionally, juglone remarkably restrained the tumor growth of LLC tumor xenografts with comparable anti-tumor efficacy to cis-platinum. Therefore, juglone may exert valuable anticancer effects and be a promising treatment option for lung cancer patients.

## Supporting information

S1 Fig (TIF)

S2 Fig (TIF)

S3 Fig (TIF)

S4 Fig (TIF)

S5 Fig (TIF)

S6 Fig (TIF)

S7 Fig (TIF)

S8 Fig (TIF)

S9 Fig (TIF)

S1 Raw data (XLSX)
