# Supplementary figures and images for: Juglone triggers apoptosis of non-small cell lung cancer through the reactive oxygen species -mediated PI3K/Akt pathway
Source: PLoS One. 2024 May 30;19(5):e0299921. doi: 10.1371/journal.pone.0299921 (PMC11139338; doi:10.1371/journal.pone.0299921)

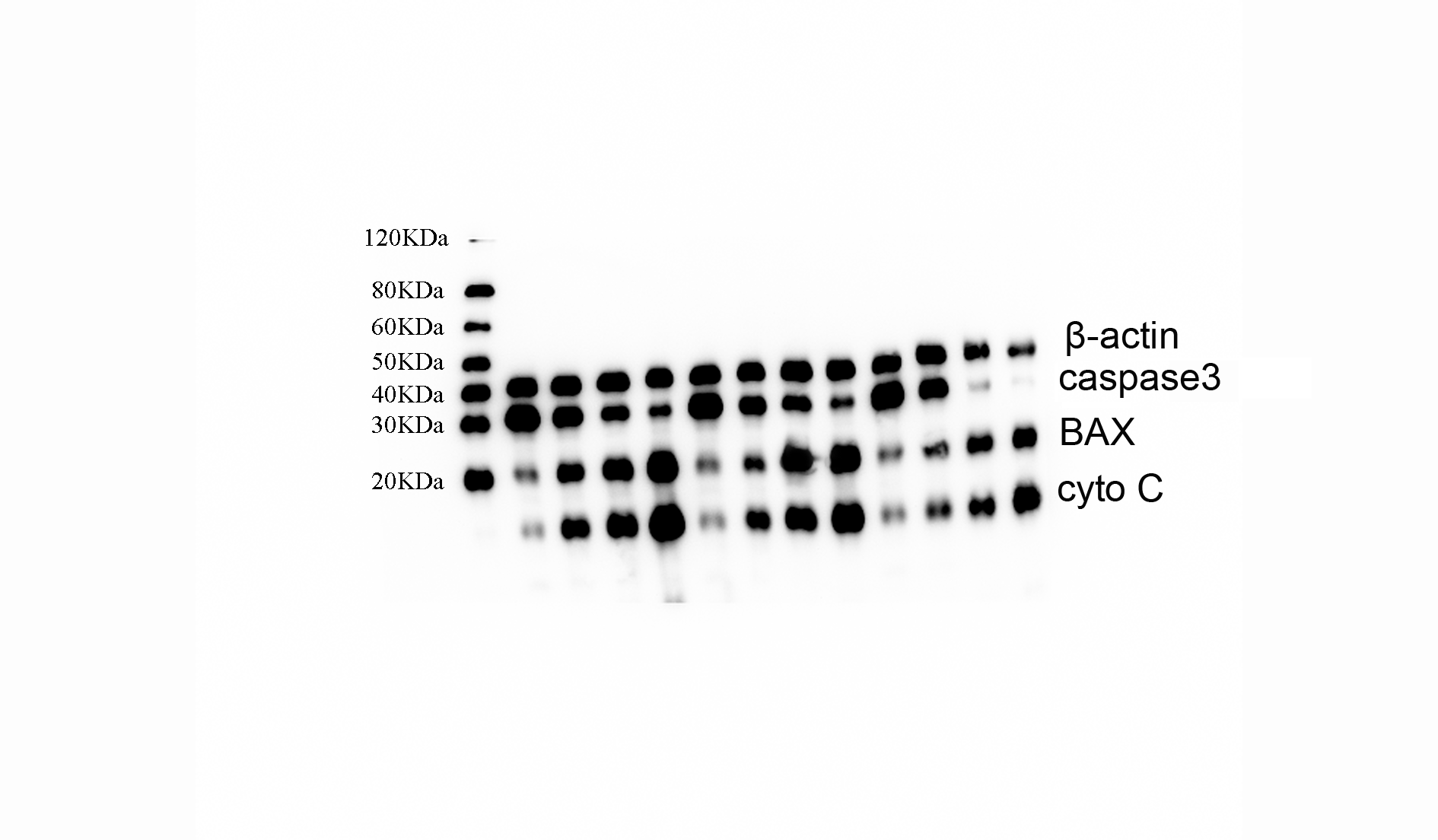

Supplement: S1 Fig — (TIF) [file pone.0299921.s001.tif]

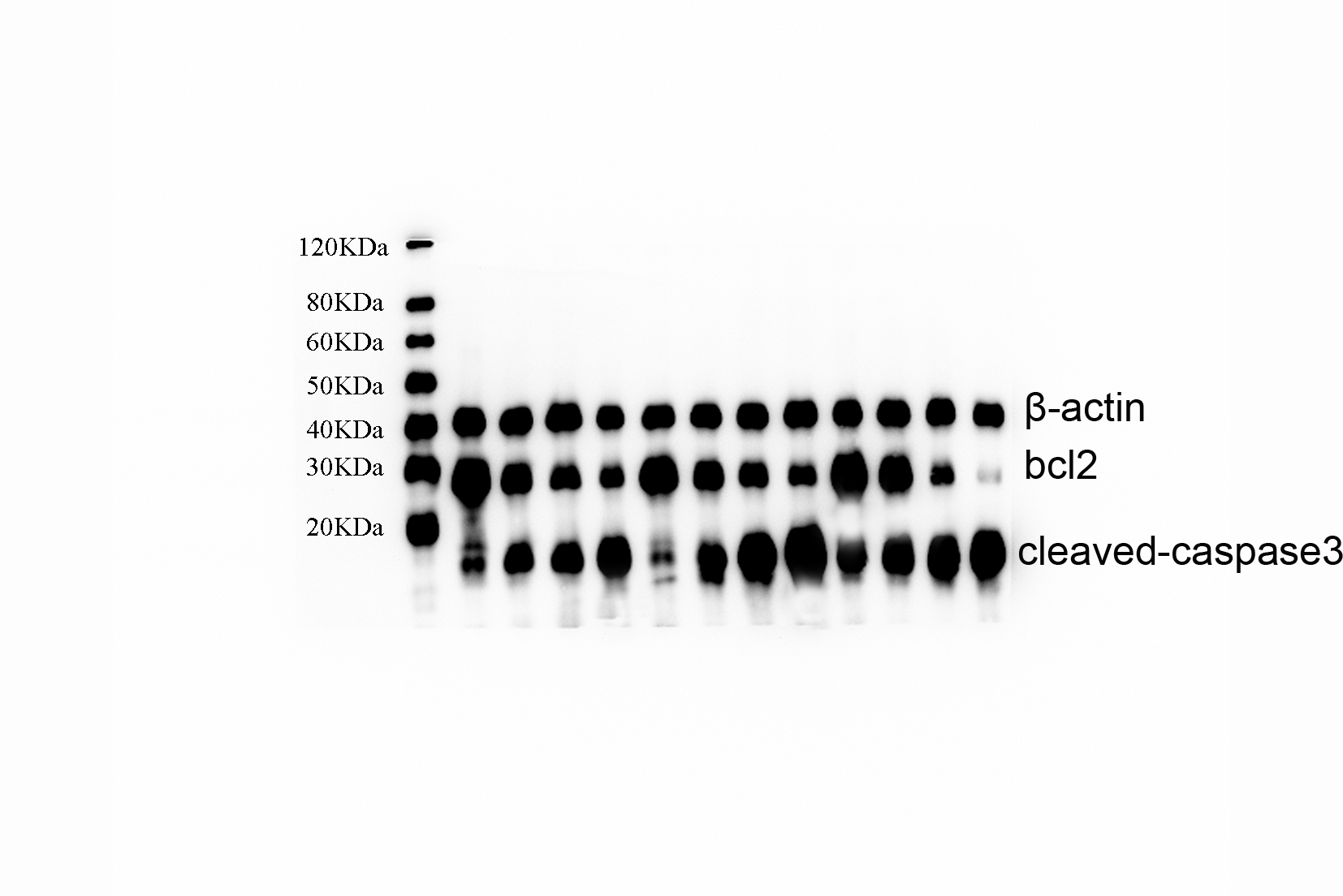

Supplement: S2 Fig — (TIF) [file pone.0299921.s002.tif]

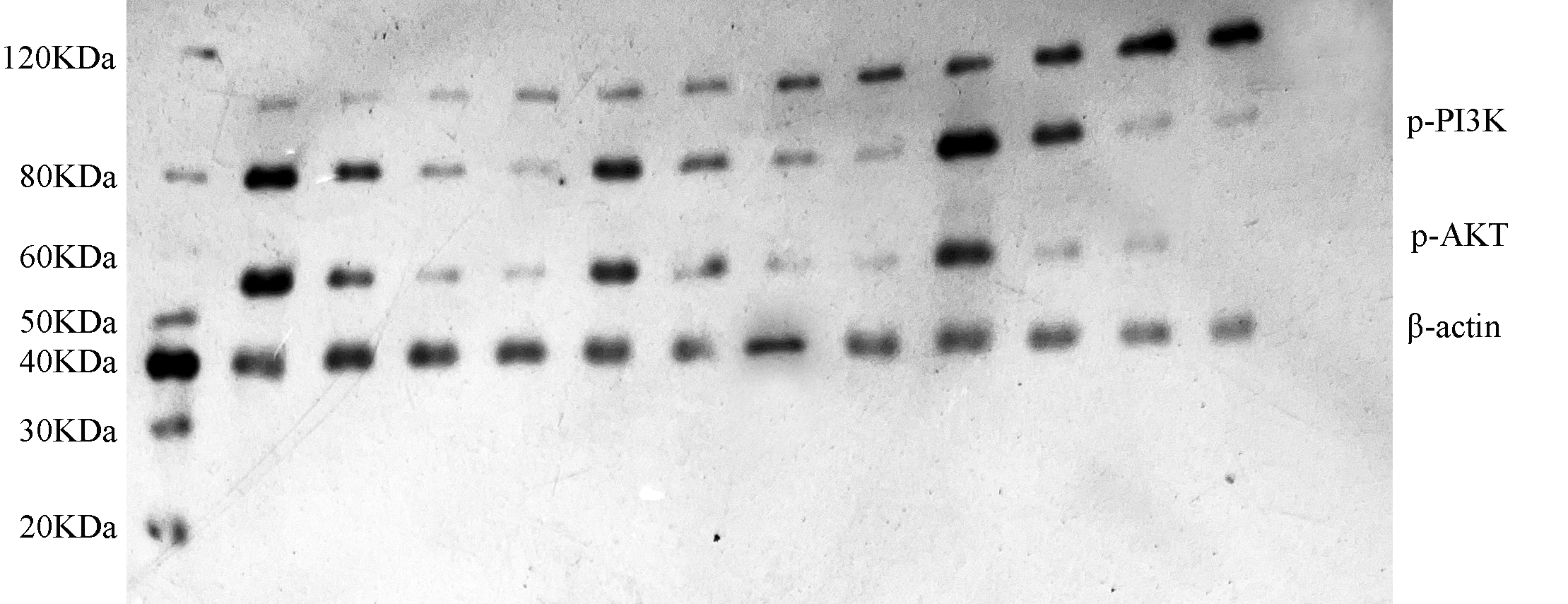

Supplement: S3 Fig — (TIF) [file pone.0299921.s003.tif]

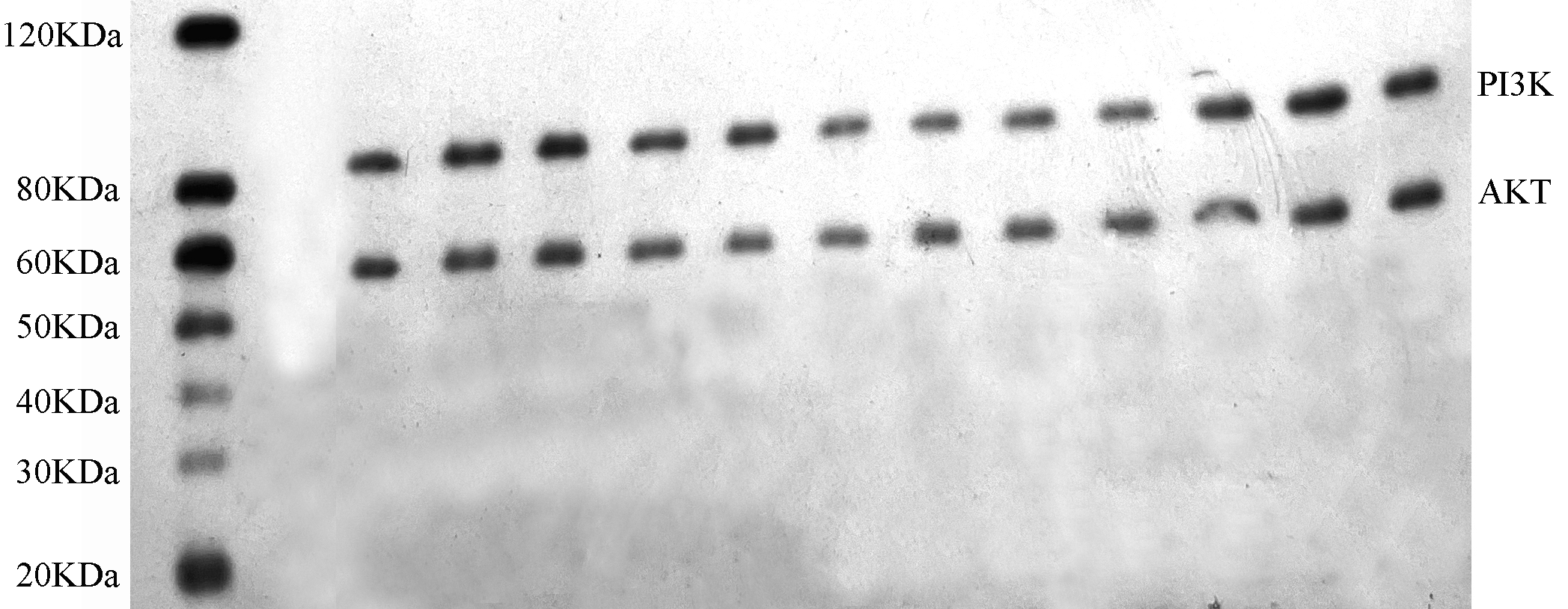

Supplement: S4 Fig — (TIF) [file pone.0299921.s004.tif]

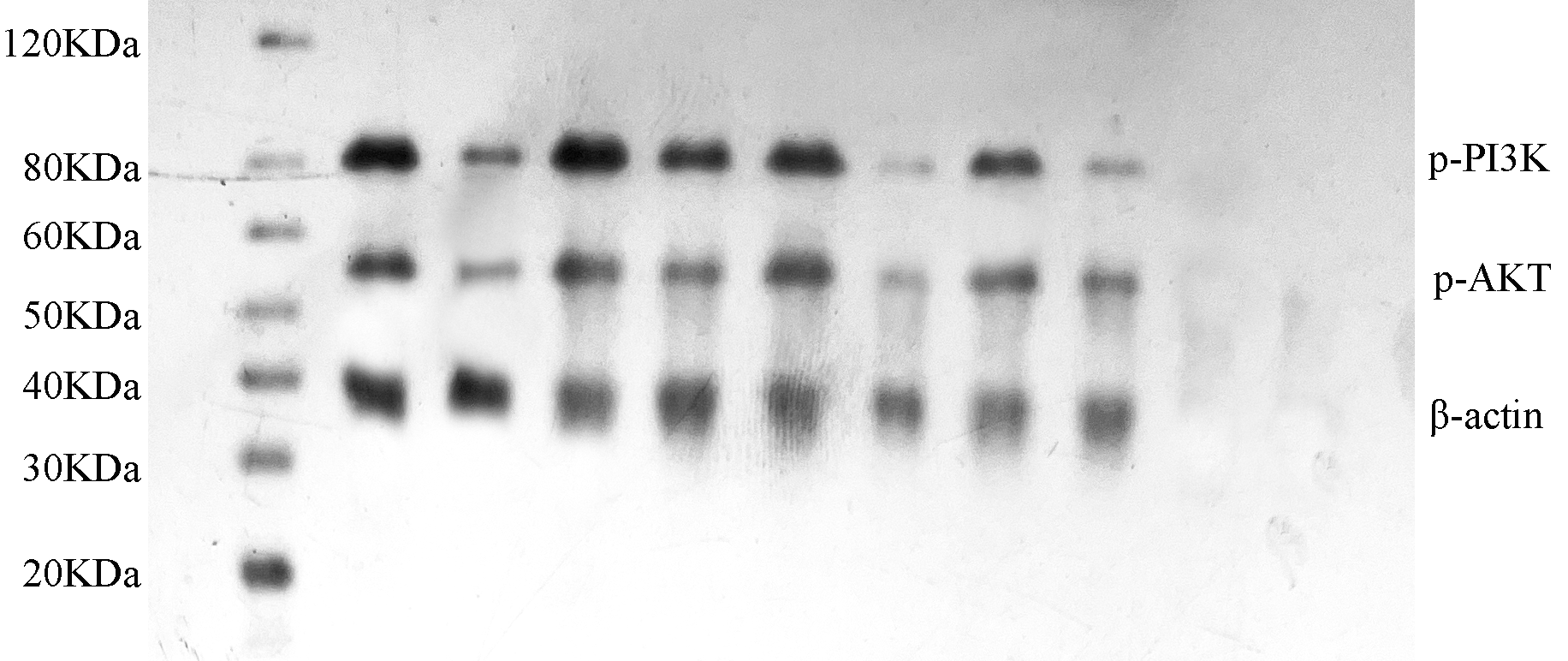

Supplement: S5 Fig — (TIF) [file pone.0299921.s005.tif]

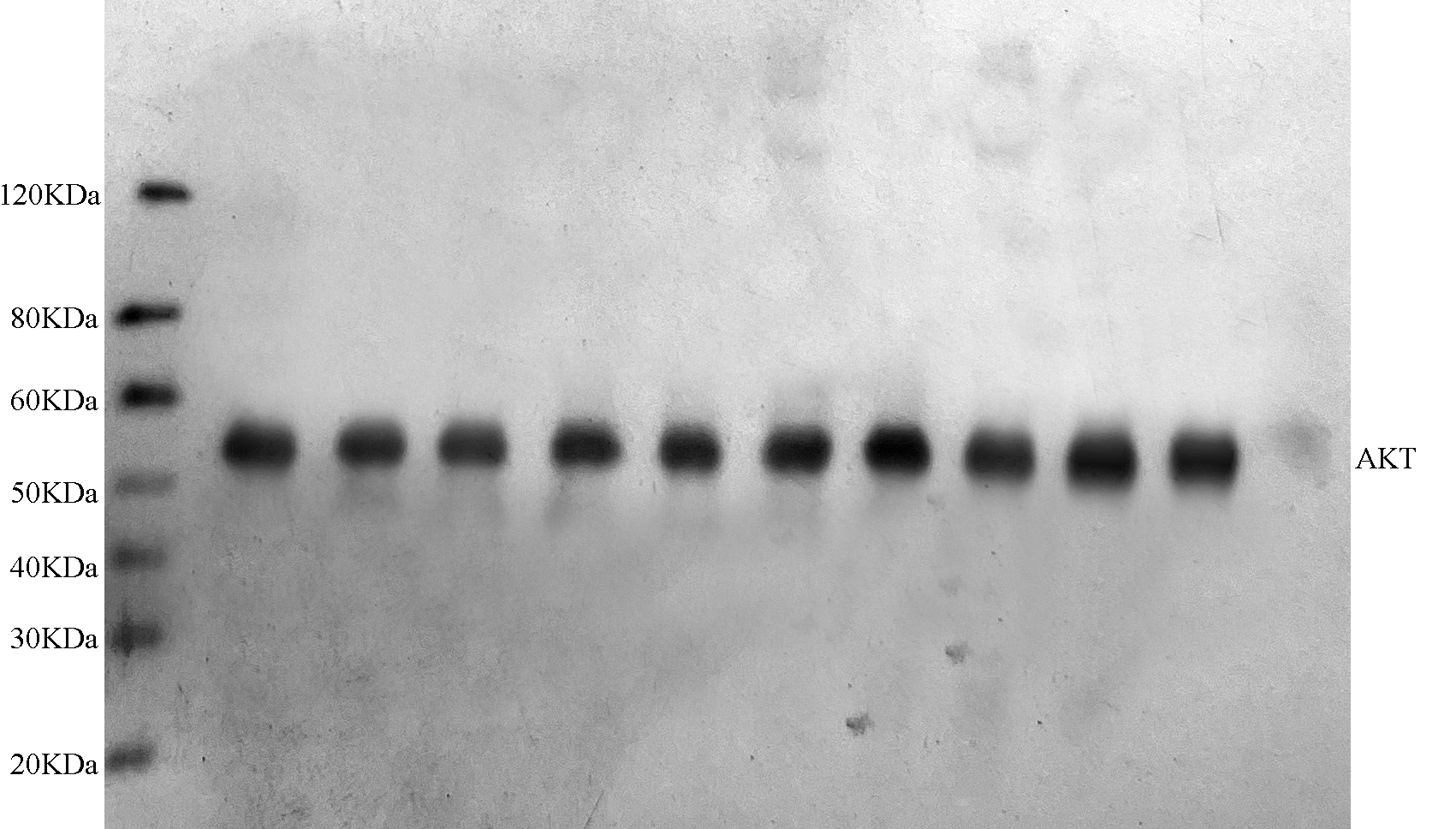

Supplement: S6 Fig — (TIF) [file pone.0299921.s006.tif]

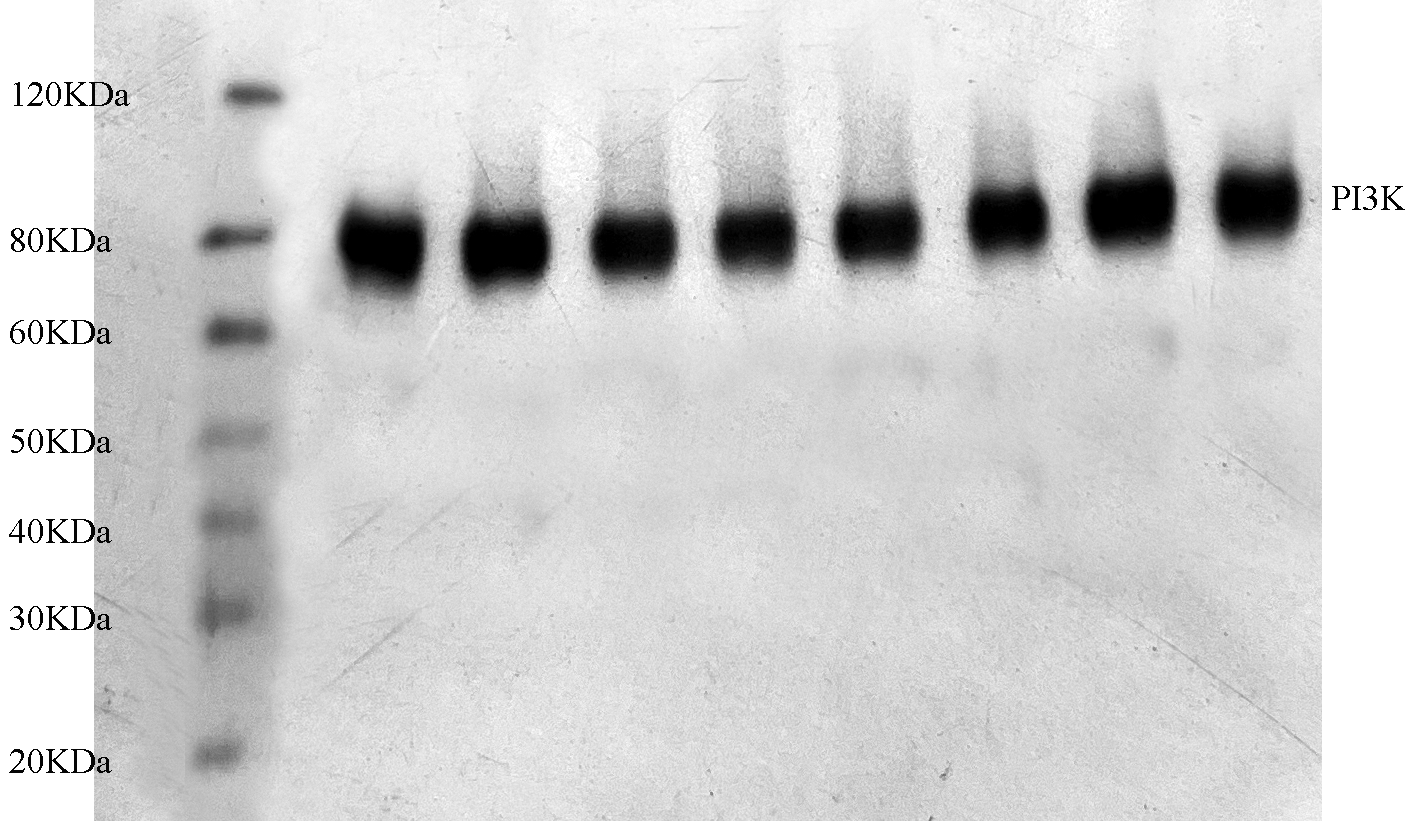

Supplement: S7 Fig — (TIF) [file pone.0299921.s007.tif]

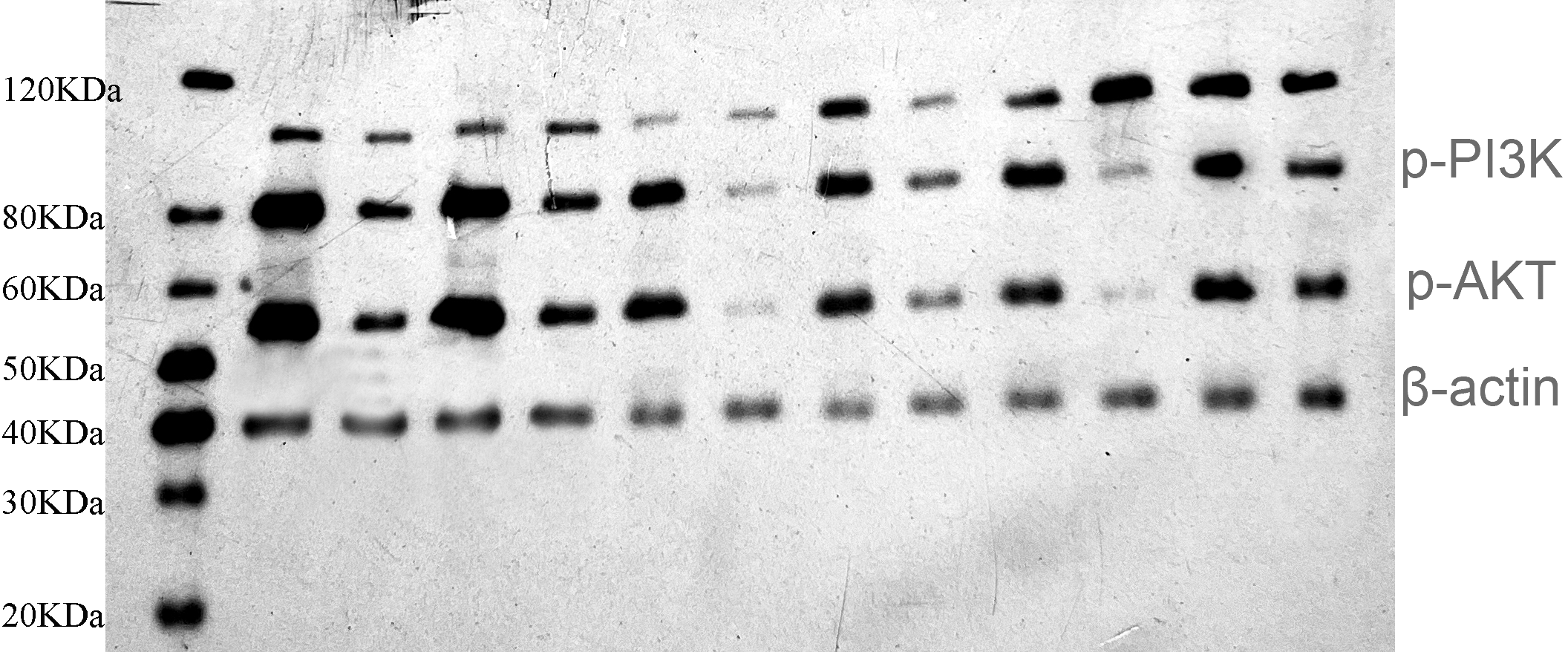

Supplement: S8 Fig — (TIF) [file pone.0299921.s008.tif]

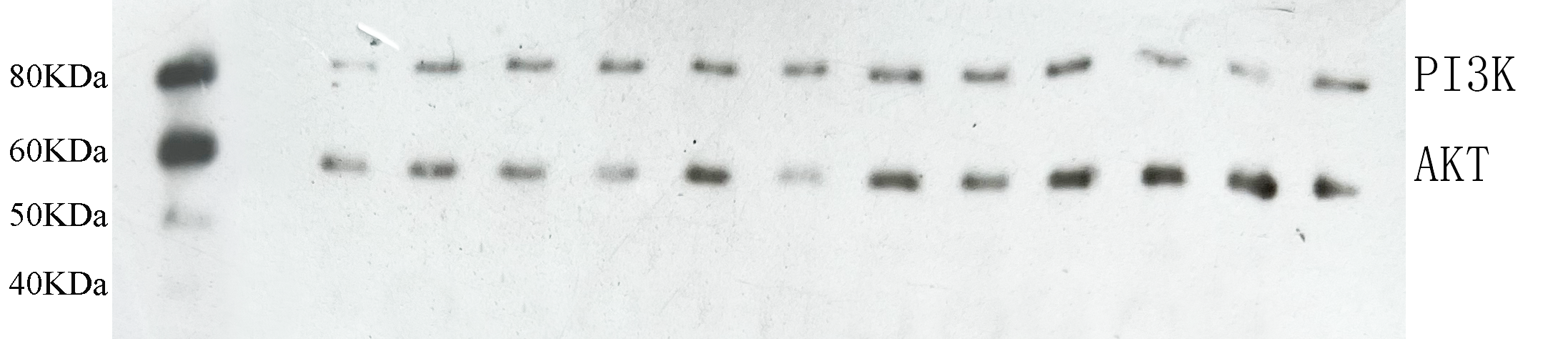

Supplement: S9 Fig — (TIF) [file pone.0299921.s009.tif]
